# The Modified Brière Equation and Its Applications

**DOI:** 10.3390/plants11131769

**Published:** 2022-07-03

**Authors:** Jun Jin, Brady K. Quinn, Peijian Shi

**Affiliations:** 1 Research Institute of Architecture, Southeast University, Nanjing 210096, China; 101010140@seu.edu.cn; 2 Biological Effects Section, St. Andrews Biological Station, Fisheries and Oceans Canada, St. Andrews, NB E5B 0E4, Canada; brady.quinn@dfo-mpo.gc.ca; 3 Bamboo Research Institute, College of Science, Nanjing Forestry University, Nanjing 210037, China

**Keywords:** axial symmetry, curve fitting, ontogenetic growth, sigmoid curve, symmetry

## Abstract

The Brière equation (BE) is widely used to describe the effect of temperature on the development rate of insects, and it can produce both symmetrical and asymmetrical bell-shaped curves. Because of its elasticity in curve fitting, the integrated form of BE has been recommended for use as a sigmoid growth equation to describe the increase in plant biomass with time. However, the start time of growth predicted by the sigmoid growth equation based on the BE is not completely comparable to empirical crop growth data. In the present study, we modified the BE by adding an additional parameter to further increase its elasticity for data fitting. We termed this new equation the modified Brière equation (MBE). Data for the actual height and biomass of 15 species of plants (with two cultivars for one species) were fit with the sigmoid growth equations based on both the BE and MBE assuming that the growth start time was zero for both. The goodness of fit of the BE and MBE sigmoid growth equations were compared based on their root-mean-square errors and the corresponding absolute percentage error between them when fit to these data. For most species, we found that the MBE sigmoid growth equation achieved a better goodness of fit than the BE sigmoid growth equation. This work provides a useful tool for quantifying the ontogenetic or population growth of plants.

## 1. Introduction

The ontogenetic growth trajectories of animals and plants usually exhibit a sigmoid pattern, and many mathematical equations have been proposed to describe the changes in growth data (e.g., biomass, diameter at breast height, height, length, etc.) with time [1,2,3,4,5,6]. Among these, the logistic equation is perhaps the most commonly used [5,6,7,8]. The three-parameter logistic equation assumes that the growth rate versus time curve is perfectly symmetrical but is not suited to all empirical datasets [9]. Thus, some equations predicting asymmetrical (skewed) growth rate curves have been used instead to reflect the growth trajectories of animals and plants [3,9,10].

In thermal biology, the development (or growth) rate (i.e., the proportion or amount of development or growth, respectively, completed per unit time) of organisms has been demonstrated to be an asymmetrical (usually left-skewed), bell-shaped function of temperature [11,12]. There are many different mathematical models that can produce skewed and symmetrical development (or growth) rate versus temperature curves [13,14]. Among these temperature-dependent development rate models, the equation proposed by Brière et al. [15] is relatively simple, yet it can produce an asymmetrical curve, includes biologically meaningful parameters, and is able to describe the effect of temperature on the development rate of many organisms:

(1) $$r(T)=ax\left(T-T_{\min}\right){\left(T_{\max}-T\right)}^{1/m},$$

where *r*(*T*) represents the development rate at temperature *T*; *T*_min_ and *T*_max_ represent the lower and upper threshold temperatures of development, respectively, and *r*(*T*) = 0 when *T* < *T*_min_ or *T* > *T*_max_; and *a* and *m* are parameters to be estimated. Brière et al. [15] suggested that a simplified version of Equation (1) could be used by fixing *m* = 2; this simplified equation, referred to as the Brière-1 equation (versus the original Brière equation, or Brière-2 equation), is not considered further herein. All subsequent references to the Brière equation hereinafter refer to the original Brière equation, or Brière-2 equation. Up to 25 May 2022, the original paper by Brière et al. [15] has been cited 745 times according to Google Scholar.

Yin et al. [3] introduced a new sigmoid growth equation using the integrated form of the beta equation while replacing temperature with time as the independent variable, and it has been shown to provide a good fit to the actual growth data of plants [3,9,16,17]. Similarly, Cao et al. [18] presented a sigmoid growth equation based on the Brière equation. However, although the new growth equation introduced by Cao et al. [18] could fit the growth data of some crops very well, the start times of growth predicted by it were not reliable as the predicted start time seriously deviated from the actual sowing time [18]. Further, when the start time is manually set to zero, the prediction errors for the sigmoid equation proposed by Cao et al. [18] relative to empirical data can be quite large. 

In this study, we propose a modification of the Brière equation by adding an extra parameter to render the new equation more flexible in curve fitting and reduce the dependency of the curve fitting on the start time of growth. We used empirical data from 15 species of plants (with two cultivars for one species) to test the validity of a new sigmoid growth equation based on this modified Brière equation (MBE) and compared the goodness of fit of the sigmoid growth equation based on the MBE to that based on the original Brière equation (BE).

## 2. Materials and Methods

### 2.1. Plant Materials

The seeds of 11 species of common agricultural crops (with two cultivars for one species, 12 crops in total), including sunflower (*Helianthus annuus*), peanut (*Arachis hypogaea*), black soybean (*Glycine max* ‘Kuromame’), soybean (*Glycine max*), kidney bean (*Phaseolus vulgaris*), garden pea (*Pisum sativum*), adzuki bean (*Vigna angularis*), mung bean (*Vigna radiata*), cotton (*Gossypium herbaceum*), sweet sorghum (*Sorghum bicolor*), corn (*Zea mays*), and Mexican corn (*Zea mexicana*), in northern China were sown in a field at Jinan, China on 27 June 2011. We then randomly sampled 20 individuals from each species (or each cultivar) to measure their dry mass on each of 15 subsequent investigation dates from 11 July to 20 September 2011 once every five days on average. In total, 3000 individual samples were collected. All individuals were sampled between 7:00 a.m. to 8:00 a.m., and the roots were washed with running water to remove soil. For small crops, whole plants were dried for 24–48 h at 60 °C. However, for large crops, such as sunflower, sweet sorghum, corn, and Mexican corn, whole plants were dried for 72 h at 80 °C. Here, we defined the crops with mean whole-plant dry mass ≥ 150 g and mean aboveground height ≥ 1.5 m on the last investigation date (i.e., 85 days from the sowing time) as large crops and those with mean whole-plant dry mass < 150 g and mean aboveground height < 1.5 m at this time as small crops. Samples were weighed after drying to obtain their dry mass. Shi et al. [5] can be consulted for further details of the sampling procedures. 

We also used the height data from four species of bamboo (*Phyllostachys iridescens*, *Phyllostachys mannii*, *Pleioblastus maculatus*, and *Sinobambusa tootsik*) previously collected at different investigation dates [9]. The bamboo shoots were grown at the Nanjing Forestry University campus in the spring of 2016. We defined the time when a bamboo shoot tip was first observed at ground level as time = 1, and the previous day as time = 0. We measured the height of each shoot at 12:00 p.m. every day at the early growth stage, and then measured the height at 12:00 p.m. once every three days when the height changed more slowly.

The above datasets were obtained from the ‘crops’ and ‘shoots’ datasets, respectively, included in the R (version 4.2.0) [19] package ‘IPEC’ (version 1.0.3) [20].

### 2.2. Methods

The original Brière equation was proposed to describe the effect of temperature on the development rate of many organisms, especially arthropods [13,15,21] (see Equation (1)). After replacing temperature *T* with time *x*, and the development rate *r*(*T*) with the growth rate *f*(*x*) in the original Brière equation, we have:

(2) $$f(x)=ax\left(x-x_{\min}\right){\left(x_{\max}-x\right)}^{1/m},$$

where *f*(*x*) represents the growth rate at time *x*; *x*_min_ and *x*_max_ represent the start and end times of growth, respectively, and *f*(*x*) = 0 when *x* < *x*_min_ or *x* > *x*_max_; and *a* and *m* are parameters to be estimated. 

Herein, we propose including an additional parameter to be estimated, δ, in Equation (2) to render it more elastic in curve fitting as follows:

(3) $$f(x)=a{\left|x\left(x-x_{\min}\right){\left(x_{\max}-x\right)}^{1/m}\right|}^{\delta }.$$

**Figure 1:**
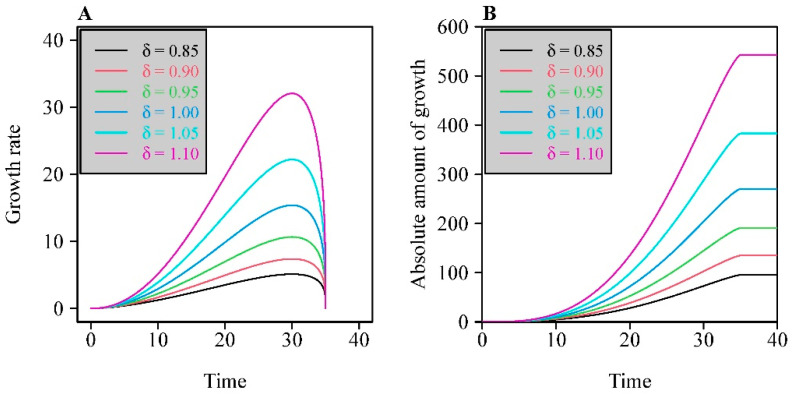
Influence of the new additional parameter, δ, on the shape of the curves plotting the modified Brière equation (**A**), and on those plotting the sigmoid equation based on the integrated form of the modified Brière equation (**B**). Here, *a* = 0.01, *m* = 3, *x*_min_ = 0, and *x*_max_ = 35. In this example, no specific units are used; the *x*-axis represents a generic time variable (hours, days, weeks, etc.) and the *y*-axes generic (**A**) growth rate (cm day^−1^, g h^−1^, etc.) and growth (cm, g, etc.) metrics.

We refer to Equations (2) and (3) as the BE and MBE for convenience hereinafter. The integrated forms of the BE and MBE were used to fit the dry mass versus time data of crops and those of height versus time data of bamboo shoots. Figure 1A illustrates the influence of the new δ parameter on the curve shapes plotted by the modified BE. 

After integrating the BE and MBE, two sigmoid growth equations are obtained, i.e.,

(4) $$y=\{\begin{array}{cc} 0 & x<x_{\min}\\ \int_{x_{\min}}^{x}f(x)\;dx & x\in \left[x_{\min},\;x_{\max}\right]\\ 0 & x>x_{\max} \end{array}.$$

Here, we obtained two sigmoid growth equations when using Equations (2) and (3) to represent *f*(*x*), respectively. There is an explicit mathematical expression for the sigmoid equation based on the integrated form of BE [3,17], which is referred to as the BE sigmoid equation hereinafter for convenience. However, there is no explicit mathematical form for the integrated form of MBE. Thus, we calculated the numerical integral of MBE, which we refer to as the MBE sigmoid equation for convenience. Figure 1B illustrates the curves produced by the MBE sigmoid equation with different δ values.

The Nelder–Mead optimization method [22] was used to fit the above equations to each plant dataset by minimizing the residual sum of squares (RSS) between observed and predicted *y* values. For the empirical crop biomass and bamboo shoot height data we used, the start time of growth is actually known. Thus, we fixed the starting time to be zero, in which case Equation (3) had four parameters and Equation (2) had three parameters. We then calculated the root-mean-square error (RMSE) for each dataset:

(5) $$\mathrm{RMSE}=\sqrt{\mathrm{RSS}/n},$$

where *n* represents the number of data points. To examine whether the additional parameter in Equation (3) was warranted relative to using the simpler Equation (2), we used the percentage error of the absolute difference (APE, in %) between the RMSE of the BE sigmoid equation and that of the MBE sigmoid equation [23,24] as follows:

(6) $$\mathrm{APE}=\frac{\left|{\mathrm{RMSE}}_{\mathrm{BE}}-{\mathrm{RMSE}}_{\mathrm{MBE}}\right|}{{\mathrm{RMSE}}_{\mathrm{BE}}}\times 100\%.$$

As a rule of thumb, when APE > 5%, the introduction of the additional parameter (δ) in Equation (3) versus (2) is justified; otherwise, it is not worthwhile. 

The whole-plant dry mass tends to be proportional to the whole-plant fresh mass [5,25] if there is no water loss. In that case, if the sigmoid equation based on the integrated form of MBE can fit the dry mass data well, it should also be applicable to fresh mass data. The difference here would only affect the numerical value of the parameter *a* in Equation (3). However, given the water loss during crop sampling, the dry mass is more reliable than the fresh mass. Thus, for the crops, we used the dry mass rather than the fresh mass to test the validities of the BE and MBE sigmoid equations.

The package ‘biogeom’ (version 1.0.5) [26,27] was used in R (version 4.2.0) [19] to estimate model parameters, and R (version 4.2.0) was also used to carry out all other calculations.

## 3. Results

**Figure 2:**
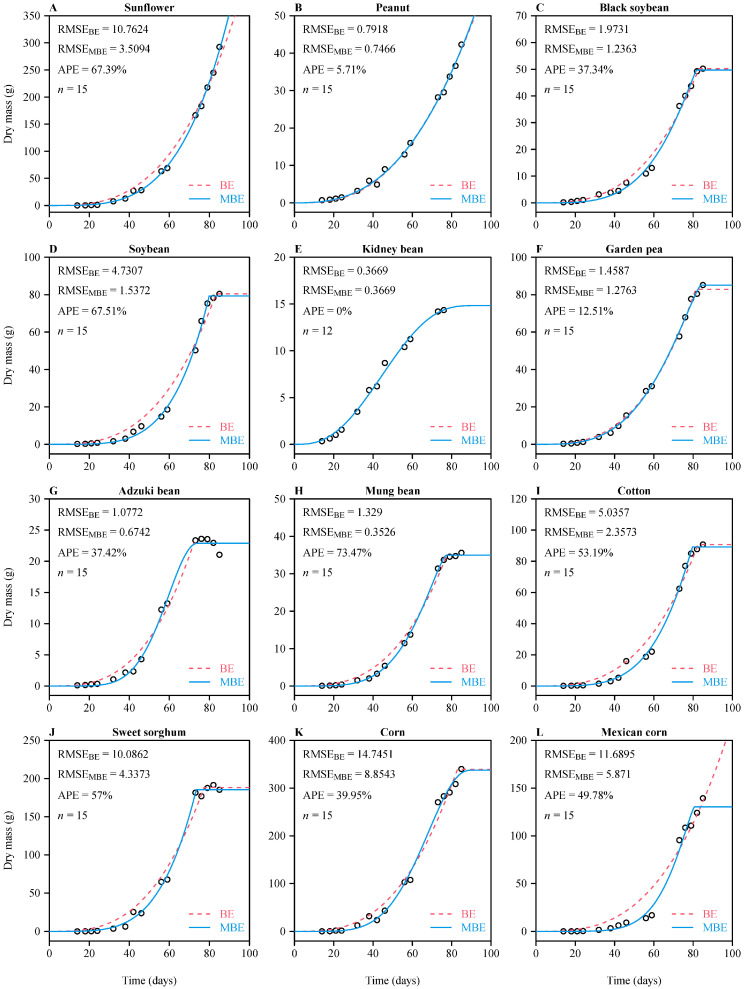
Results of fitting two sigmoid functions (based on the BE and MBE) to the whole-plant dry mass versus time data of 11 crop species (with two cultivars for one crop species). The small open circles represent the observed dry mass at different times after the sowing date; the red dashed curves represent the dry mass values predicted by the BE sigmoid equation; the blue solid curves represent the dry mass values predicted by the MBE sigmoid equation. RMSE represents the root-mean-square error between the observed and predicted *y* values; APE represents the percentage error of the absolute difference between the two equations’ RMSE values; *n* represents the sample size. Panels (**A**–**L**) represent different crops.

For each of the crop species tested except kidney bean (11/12 datasets), the MBE sigmoid equation had a lower RMSE than the BE sigmoid equation, and the APE was greater than 5% (Figure 2). This confirmed the validity of the MBE sigmoid equation and its superiority relative to the BE sigmoid equation. Because of the lack of biomass data at the mature stages of sunflower and peanut, both sigmoid functions overestimated the end time of growth, leading the predicted curves to exhibit exponential growth at the early and middle growth stages (Figure 2A,B). 

**Figure 3:**
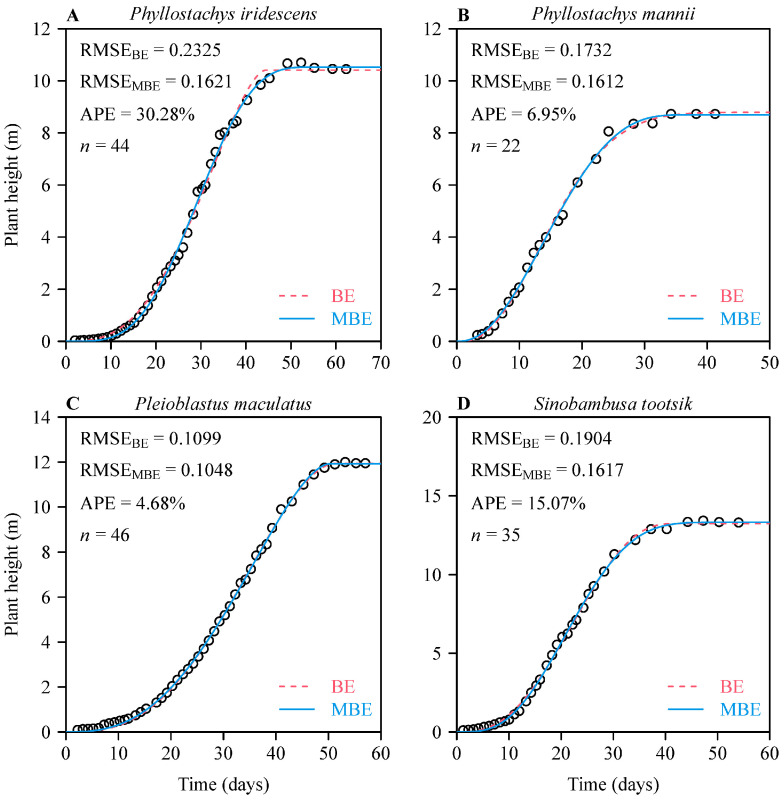
Results of fitting two sigmoid functions (based on the BE and MBE) to the shoot height versus time data of four species of bamboo. The small open circles represent the observed shoot height at different times (days) after the date when the shoot tip first emerged from the soil; the red dashed curves represent the height values predicted by the BE sigmoid equation; the blue solid curves represent the height values predicted by the MBE sigmoid equation. RMSE represents the root-mean-square error between the observed and predicted *y* values; APE represents the absolute percent error difference between the two equations’ RMSE values; *n* represents the sample size. Panels (**A**–**D**) represent different bamboo species.

For each of the four species of bamboo considered, the MBE sigmoid equation also had a lower RMSE than the BE sigmoid equation, and the APE was larger than 5% for three of the four species (Figure 3). For *Pleioblastus maculatus*, the APE was equal to 4.68%, or approximately 5%. These results demonstrate that the BE and MBE sigmoid equations are both suited to representing data for growth in terms of height for bamboo shoots, at least provided that the initial time at which growth starts (i.e., *x* = 0) can be accurately known. Nevertheless, the MBE sigmoid equation is still better.

The estimated values of the parameters of the BE sigmoid equation and the MBE sigmoid equation for the 15 species of plants (with two cultivars for one species) tested are listed in Tables S1 and S2 in the online Supplementary Materials.

## 4. Discussion

### 4.1. Elasticity in Curve Fitting of the Two Sigmoid Equations

The inclusion of an additional parameter, δ, in the MBE greatly increased the elasticity of the MBE sigmoid equation in curve fitting; i.e., it increased the range of sigmoid curves with different curvatures that can be fit relative to the BE sigmoid equation (Figure 1). The larger the numerical value of δ is, the greater the curvature of the line will be that is generated by the MBE sigmoid equation. For 14 of the 16 tested datasets, the APE values were greater than 5%, which indicates that the increased elasticity in curve fitting achieved by the additional parameter in the MBE sigmoid equation outweighed the cost of the model’s increased complexity [28]. In fact, the elasticities of the two sigmoid equations in curve fitting mainly depend on the abilities of the BE and MBE to accurately describe growth rates. However, it is more difficult to directly measure the growth rate per unit time in practice than to measure accumulated biomass or height after some time interval has elapsed. Although Cao et al. [18] showed that the BE sigmoid growth function achieved a good fit to the dry mass data of six of the twelve datasets of crops used herein, the estimated growth start times were later than the actual sowing time and the times seedings appeared (see Figure 1 in Ref. [18]). It is apparently reasonable to fix the start time to the sowing time given that all the seeds were planted on the same day. In our study, the MBE sigmoid function achieved a good fit to observations without estimating unreasonable start times, and it showed good elasticity in curve fitting through changes in the numerical value of the δ parameter. After all, the *x*_min_ parameter of the BE and MBE has an explicit geometrical and biological meaning, so it is unreasonable to over- or underestimate its numerical value. An over- or underestimated *x*_min_ value can easily mislead the user of such equations to predict inaccurate growth. On the other hand, the new δ parameter has no clear biological or geometrical meaning, and it only serves as a constant to be estimated to improve the flexibility of the equation in curve fitting.

### 4.2. Why We Did Not Compare the Two Sigmoid Equations with Other Equations

As stated in the Introduction section, there are many temperature-dependent development (or growth rate) equations that can produce a skewed curve [9,13,14,21]. Shi et al. [17] found that the sigmoid equation based on the integral of the beta equation [3] can achieve better goodness of fit than traditional growth equations, including the exponential and logistic equations [8], Gompertz equation [29], von Bertalanffy equation [1], and ontogenetic growth equation [2]. Shi et al. [9] demonstrated that the sigmoid equation based on the integral of the modified beta equation and that of the modified Lobry–Rosso–Flandrois (LRF) equation [30,31] can be better-suited to representing the ontogenetic growth data of animals and plants. However, the mathematical expressions of the BE and MBE are simpler than those of the beta equation, modified beta equation, LRF equation, and modified LRF equation (Equations (2) and (3) versus those published in Ref. [9]). In the present study, we did not compare the BE and MBE sigmoid equations with other sigmoid equations because we mainly wanted to examine the strengths and drawbacks of the BE and MBE sigmoid equations and to test whether the latter is better than the former for curve fitting. A systematic comparison among sigmoid equations is worth carrying out using more datasets in future studies. 

### 4.3. Reliability of Estimated Parameters in the Two Sigmoid Equations

Although the estimated parameters, especially the start and end times of growth, are considered to be meaningful for predicting when these time points will occur during the growth of plants, the reliability of the prediction is largely constrained by whether the dataset includes a full range of growth data, especially those at the mature stage (i.e., the asymptotic values of plant biomass or height). If the asymptotic values of plant size at the mature stage are known, the predicted end time of growth tends to be reliable [6,9,18]; however, if such data are lacking, the predicted end time tends to be overestimated (see Figure 2A,B,L). To reduce the uncertainty of predictions and the complexity of the model, we suggest fixing the starting time and allowing the δ parameter to vary. It is more important to estimate the end time rather than the start time of growth for a crop product or forest resource management. If the estimated end time of growth is reliable, it can produce a reliable growth rate curve with which one can predict when plants reach their maximum growth rate.

### 4.4. Other Potential Applications of the Modified Brière Equation

**Figure 4:**
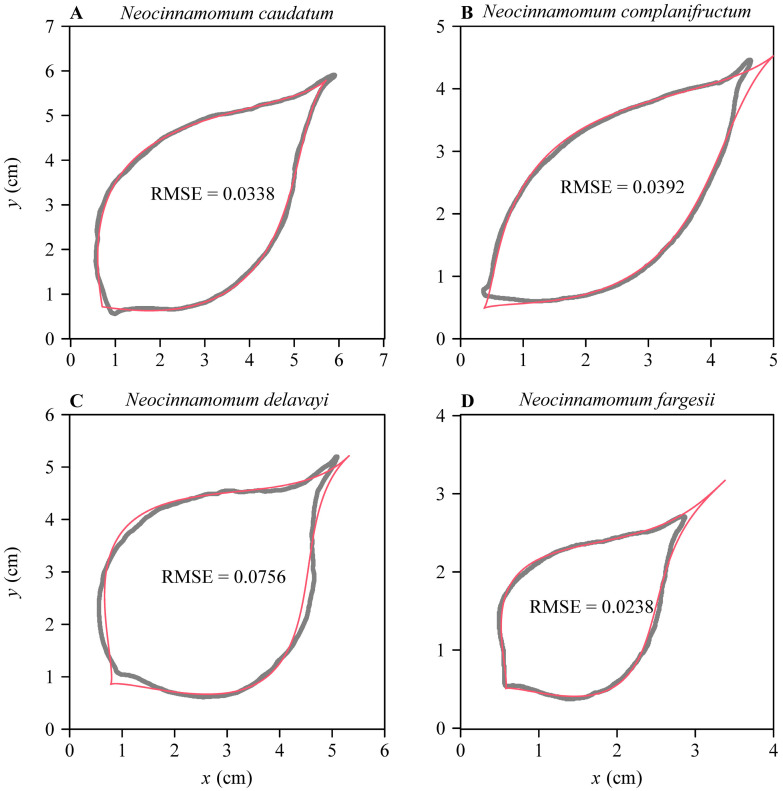
Results of fitting the MBE to the boundary coordinates of the leaves of four species of *Neocinnamomum*. The gray curves are scanned (actual) leaf perimeters, and the red curves are leaf perimeters predicted by the MBE. The boundary coordinate data came from the dataset ‘*Neocinnamomum*’ in R package ‘biogeom’ (https://cran.r-project.org/web/packages/biogeom/index.html; accessed on 30 May 2022).

Given that the MBE is flexible in fitting a skewed bell-shaped curve, it is potentially useful to model the profiles of ovate leaves [32]. Shi et al. [32] formed two axially symmetrical curves using the modified beta equation (also the modified LRF equation) to fit the ovate leaf shapes of *Neocinnamomum* plants (Lauraceae). Given that the MBE can generate similar skewed curves, the MBE should be similarly useful in describing ovate leaf shape. Relative to the BE, the MBE is more flexible due to its inclusion of an additional parameter, δ, as discussed above, and it can thus also fit the actual boundary coordinates of ovate leaves (Figure 4). Like the original BE, the MBE may also be useful in data-fitting and modelling of temperature-dependent development and growth rates in animals and plants [15,21]. While such additional applications of the MBE are beyond the scope of the present work (and, thus, we do not discuss them in detail here), it will be worthwhile for future studies to assess the validity of the MBE in fitting ovate and obovate leaf shapes and temperature-dependent development rate data.

## 5. Conclusions

A better goodness of fit was obtained with the MBE sigmoid equation for 15 of the 16 datasets of plants than the BE sigmoid equation, and it had the same RMSE as that of the BE sigmoid equation for the one remaining species. For most datasets (14/16), the percentage errors of the absolute difference between the RMSE of the MBE sigmoid equation and that of the BE sigmoid equation were greater than 5%, which indicates that the addition of the δ parameter to the original Brière equation was worthwhile, improving the validity of the MBE sigmoid equation in reflecting the actual growth data of plants. In addition, by virtue of the estimated model parameters, whether the growth curve of a given species is left- or right-skewed can be evaluated (i.e., based on the derivative of the MBE sigmoid equation), and, thus, one can use this to predict the time associated with the maximum growth rate.

## Data Availability

The data used in the present work have been packaged in package ‘IPEC’ (version 1.0.3; https://cran.r-project.org/web/packages/IPEC/index.html; accessed on 24 May 2022). The crop biomass growth data were tabulated in dataset ‘crops’, and the bamboo height growth data were tabulated in dataset ‘shoots’.

## Supplementary Materials

The following supporting information can be downloaded at: https://www.mdpi.com/article/10.3390/plants11131769/s1, Table S1: The estimates of the model parameters for the whole-plant dry mass versus time data of 12 crops; Table S2: The estimates of the model parameters for the shoot height versus time data of four species of bamboo.
